# Supplementary material for: Impact of Active Physiotherapy Rehabilitation on Pain and Global and Functional Improvement 1–2 Months after Lumbar Disk Surgery: A Systematic Review and Meta-Analysis
Source: Healthcare (Basel). 2022 Oct 5;10(10):1943. doi: 10.3390/healthcare10101943 (PMC9601491; doi:10.3390/healthcare10101943)
Supplement: Supplementary file 1 [file healthcare-10-01943-s001.zip › healthcare-1885864-supplementary.pdf]

Supplementary Table S1. Studies obtained from every database.

PubMed/MEDLINE	Web of Science	Scopus	CINAHL Plus	Cochrane
278	442	489	158	27
